# Cytocompatibility of pH-sensitive, chitosan-coated Fe_3_O_4_ nanoparticles in gynecological cells

**DOI:** 10.3389/fmed.2022.799145

**Published:** 2022-07-22

**Authors:** Taohong Zhang, Lisha Wang, Xinyi He, Hailin Lu, Li Gao

**Affiliations:** ^1^Department of Obstetrics and Gynecology, The First Affiliated Hospital of Xi’an Jiaotong University, Xi’an Jiaotong University, Xi’an, China; ^2^College of Mechanical and Electronic Engineering, Xi’an Polytechnic University, Xi’an, China

**Keywords:** chitosan-coated Fe_3_O_4_ (CS-Fe_3_O_4_) nanoparticles, gynecological cells, cytocompatibility, ovarian cancer, gestational choriocarcinoma

## Abstract

Nanoparticles that contact human cells without damaging basic human tissues are becoming more widely used in medicine. Efficient delivery to the intracellular target cell or compartment through the cell membrane must be achieved with minimal cytotoxicity to healthy cells. Fe_3_O_4_ nanoparticles have been widely used in biomedical research for their magnetic, non-toxic, and biocompatible properties. However, the effects of Fe_3_O_4_ nanoparticles coated with chitosan (CS) on gynecological cells are unclear. In this study, the Fe_3_O_4_ nanoparticles were coated with CS to enhance their cytocompatibility and dispersion in water. These CS-Fe_3_O_4_ nanoparticles were taken up by gynecological cells and did not affect cell viability *in vitro*. They have greater cytocompatibility in acidic environments than normal Fe_3_O_4_ nanoparticles and have the potential for drug delivery into gynecological cells.

## Introduction

Gynecological cancers such as cervical cancer, endometrial carcinoma, ovarian cancer, and gestational choriocarcinoma are the most common malignant diseases. They are treated by different methods, namely, surgery, radiotherapy, multichemotherapy, immunotherapy, and/or targeted therapy (1, 2). However, current treatments often negatively impact the health, fertility, and even the lifespan of women. Immunotherapy typically involves vascular endothelial growth factor, PD-1/programmed death ligand 1, tyrosine kinase, or other targeting monoclonal antibodies despite some patients not showing improvement and exhibiting serious side effects (3–7). Therefore, it is necessary to look for alternative biocompatible materials for the diagnosis, evaluation, and treatment of gynecological cancers.

In recent years, many experiments and simulation studies have focused on the influence of the physical and chemical properties of nanoparticles on cellular interactions (8–12). Superparamagnetic iron oxide materials are widely used in drug delivery, medical imaging, cell targeting, and hyperthermia therapy (13–17). Superparamagnetic Fe_3_O_4_ (magnetite) material is usually used in cancer hyperthermal treatment. It is of particular interest because it has far fewer side effects than chemotherapy or radiotherapy (18). Magnetite nanoparticles coated with polyarabic acid and carrying doxorubicin showed outstanding membrane permeability, excellent drug loading and release behavior, minimal *in vivo* toxicity, and promising therapeutic potential (19). The surface coating of nanoparticles also affects the interface aggregation of nanoparticles (20, 21), however, the effect on cellular uptake is unclear. The calculation model of hyperthermia has been widely used in the study of magnetite (Fe_3_O_4_), maghemite (Fe_2_O_3_), and various gold nanomaterial shapes (22, 23). Size-dependent nanoparticle cellular uptake through endocytosis is crucial for drug delivery in nanomedicine and a simulation of their membrane interaction may predict efficacy (24). The passive endocytosis efficiency of spherical particles is high, whereas the endocytosis of particles with sharp edges is inhibited (25).

Fe_3_O_4_ nanoparticles with a core diameter under 20 nm are usually used as tools for the diagnosis and treatment of tumors since they have unique magnetic responsiveness and photothermal effects (26–29). They are the preferred nanomaterial for MRI contrast agents since they have a strong *T*2 relaxation signal (30) and are safe for use in humans due to the efficient biodegradation of the free ions (31–33). Fe_3_O_4_ nanoparticles require appropriate surface functionalization to prevent them from being cleared from the circulatory system by the immune system (34). Chitosan (CS) is the second-largest natural cationic polysaccharide and is renewable, biodegradable, non-toxic, and has biocompatible properties (35–38). CS has abundant functional groups on its surface; thus, CS-Fe_3_O_4_ nanoparticles can interact with other molecules and potentially deposit a variety of inorganic and organic materials *in vivo*. In this study, the sol–gel method was successfully used to prepare CS-Fe_3_O_4_ nanoparticles. As a result, the Fe_3_O_4_ nanoparticles were wrapped by CS. Their biocompatibility is greater than that of inorganic nanoparticles, which reflects their appreciable clinical application potential (39, 40). In addition, CS has abundant functional groups on its surfaces, meaning that the CS-Fe_3_O_4_ nanoparticles are able to interact with other molecules and can easily deposit a variety of inorganic and organic materials.

In this study, CS-Fe_3_O_4_ nanoparticles were analyzed using scanning electron microscopy (SEM), transmission electron microscopy (TEM), Fourier transform infrared (FT-IR) spectroscopy, X-ray photoelectron spectroscopy (XPS), thermogravimetric analysis (TG), and differential scanning calorimetry (DSC). Then, the methyl thiazolyl tetrazolium (MTT) method was used to test the toxicity of gynecological cells, and cell viability data were measured. Due to their low toxicity properties, these CS-Fe_3_O_4_ nanoparticles may have excellent performance in the encapsulation and release of drugs, in nanoparticle targeting, medical imaging, cell targeting, and hyperthermia therapy.

## Materials and methods

### Materials

FeCl_3_⋅6H_2_O and ammonia solution (25%) were purchased from Damao Chemical Reagent Factory, Tianjin, China. FeCl_2_⋅4H_2_O was from Tianjin Guangfu Fine Research Institute, Tianjin, China. Acetic acid was from Fuyu Fine Chemical Co., Ltd., Tianjin, China. CS (degree of deacetylation ≥90%; molecular weight, 700–800 kDa) was purchased from Shanghai Lanji Technology Co., Ltd. (Shanghai, China). All materials used in the research were of analytical reagent grade.

### Cell culture

The SKOV-3 adherent human ovarian cancer cell line and the JEG-3 human choriocarcinoma cell line were obtained from the Shanghai Cell Bank of the Chinese Academy of Sciences (Shanghai, China). The CAOV-3 human ovarian cancer cell line was purchased from Procell Life Science & Technology Co., Ltd. The A2780 human ovarian cancer cell line was obtained from the Shandong Academy of Medical Sciences (Jinan, China). The human first-trimester extravillous trophoblast cell line (HTR-8) was obtained from the American Type Culture Collection (Manassas, VA, United States), and the JAR human choriocarcinoma cell line was obtained from the Beijing Cell Bank of the Chinese Academy of Sciences (Beijing, China). HTR-8, JAR, and A2780 cells were cultured in RPMI-1640 medium with 10% fetal bovine serum and an antibiotic solution (100 U/ml penicillin and 100 μg/ml streptomycin) at 37°C in a 5% CO_2_ atmosphere, and JEG-3, SKOV-3, and CAOV-3 cells were cultured in Dulbecco’s Modified Eagle Medium (DMEM; Gibco, Grand Island, NY, United States) with 10% fetal bovine serum and an antibiotic solution (100 U/ml penicillin and 100 μg/ml streptomycin) at 37°C in a 5% CO_2_ atmosphere. The cell experiments were carried out in the Department of Obstetrics and Gynecology, The First Affiliated Hospital of Xi’an Jiaotong University, China.

RPMI-1640 medium or DMEM medium with 10% fetal bovine serum was prepared at different pH values by the addition of acetic acid or sodium bicarbonate to obtain pH values of 5.5, 6, 6.5, 7, or 7.5 followed by filtering with 0.22-micron diameter filters (Millipore, MA, United States) into sterile glass bottles.

### Preparation of CS-Fe_3_O_4_ nanoparticles

In total, 1 g of ferric chloride hexahydrate and 0.5 g of ferrous chloride tetrachloride were dissolved in 50 ml of deionized water. Then, 50 ml of 1% w/v CS sol was prepared by dissolving CS in 1% w/v acetic acid. The iron ion solution was added to the CS sol, and the temperature was increased to 75°C under a magnetic field. Ammonia solution was added with stirring until the solution turned black and stirring was continued for 0.5 h. The precipitate was centrifuged at 3,750 g for 30 min and then repeatedly washed with deionized water until a neutral pH was obtained. Finally, CS-Fe_3_O_4_ nanoparticles were lyophilized. Fe_3_O_4_ nanoparticles were prepared using the above method described without CS addition.

### Characterization of CS-Fe_3_O_4_ nanoparticles

Fourier transform infrared spectroscopy (FT-IR) was performed using a Nicolet iS50 spectrometer (Thermo Fisher Scientific, MA, United States). XPS was performed with an ESCALAB 250 Xi^+^ XPS instrument (Thermo Fisher Scientific, MA, United States). X-ray diffraction (XRD) was performed with a D8 Advance system (Bruker, Germany). TG and DSC analyses were performed under an oxygen atmosphere using a simultaneous thermal analyzer 409 CD (Netzsch-Gerätebau GmbH, Germany). CS-Fe_3_O_4_ nanoparticles were thoroughly dissolved in 1% w/v acetic acid and observed with an H-7650 transmission electron microscope (Hitachi, Tokyo, Japan). The morphology of Fe_3_O_4_ and CS-Fe_3_O_4_ nanoparticles was determined by SEM with a SU3500 instrument (Techcomp, Shanghai, China).

### Cytocompatible tests

#### Methyl thiazolyl tetrazolium assay

A modified MTT [3-(4,5-dimethylthiazole-2-yl)-2,5-diphenyltetrazolium bromide] assay was performed to measure cell viability according to the manufacturer’s instructions. In brief, HTR-8, JAR, A2780, and SKOV-3 cells were seeded at a density of 4 × 10^3^ cells per well in 96-well plates, while JEG-3 and CAOV-3 cells were seeded at a density of 6 × 10^3^ cells per well, then treated with different concentrations of Fe_3_O_4_ and CS-Fe_3_O_4_ (0–10 μg/ml) for 24 and 48 h, respectively. In addition, SKOV-3 and CAOV-3 cells were incubated with Fe_3_O_4_ and CS-Fe_3_O_4_ (10 μg/ml) at different pH values for the same duration. Then, 20 μl MTT (5 mg/ml) was added to 180 μl complete medium per well and incubated for 4 h. The medium was removed, then 130 μl of DMSO was added to each well, the plates were shaken for 10 min, and the absorbance at 490 nm was measured using an xMark microplate absorbance spectrophotometer (Bio-Rad, Hercules, CA, United States). The experiments were conducted in triplicate and the values are expressed as the mean ± standard error.

#### Flow cytometry analysis

SKOV-3 cells (5 × 10^4^) were placed in 500 μl of complete media seeded into 6-well plates and treated with Fe_3_O_4_ and CS-Fe_3_O_4_ (10 μg/ml) at different pH values for 48 h before performing cell cycle arrest analysis. The cells were harvested *via* centrifugation at 1,000 rpm for 5 min at room temperature, washed with ice-cold phosphate-buffered saline (PBS; 137 mM NaCl, 2.7 mM KCl, 10 mM Na_2_HPO_4_, and 2 mM KH_2_PO_4_ pH 7.2) three times, then fixed with 70% ethanol at 4°C overnight. Fixed cells were treated with 100 μg/ml propidium iodide (PI) and 100 μg/ml RNase A in PBS and incubated at room temperature for 25 min. Finally, cell cycle arrest was analyzed using a FACSCalibur cell sorter (BD Biosciences, Franklin Lakes, NJ, United States) and CellQuest software version 3.3 (BD Biosciences) according to the manufacturer’s instructions.

#### Calcein-AM/PI staining for viable and dead cells

SKOV-3 cells (2 × 10^4^) were placed in 500 μl of complete media, seeded into 24-well plates, and treated with Fe_3_O_4_ and CS-Fe_3_O_4_ (10 μg/ml) at different pH values for 24 h. The culture medium was removed, gently washed two to three times with PBS, and stained with assay solution (2 μl/ml calcein-AM and 1 μl/ml PI) according to the manufacturer’s instructions (MoBiTec GmbH, Göttingen, Germany) at 37°C for 15 min. The digital images of viable cells (green fluorescence, excitation wavelength: 490 nm, emission wavelength: 515 nm) and dead cells (red fluorescence, excitation wavelength: 535 nm, emission wavelength: 617 nm) were visualized using a fluorescence microscope. This experiment was performed in triplicate.

The cell experiments were carried out at the First Affiliated Hospital of Xi’an Jiaotong University, China.

## Results and discussion

### Scanning electron microscopy and transmission electron microscopy analyses

**FIGURE 1:**
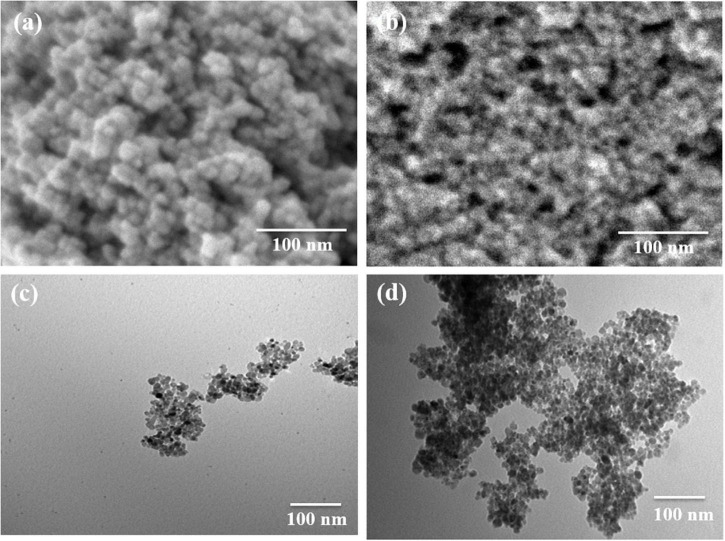
Morphology of Fe_3_O_4_ and CS-Fe_3_O_4_ nanoparticles. **(a)** High-resolution scanning electron microscopy (SEM) image of Fe_3_O_4_ nanoparticles. **(b)** High-resolution SEM image of CS-Fe_3_O_4_ nanoparticles. **(c)** Transmission electron microscopy (TEM) image of Fe_3_O_4_ nanoparticles. **(d)** TEM image of CS-Fe_3_O_4_ nanoparticles.

The synthesized Fe_3_O_4_ nanoparticles show an apparent resemblance to small balls of aggregates (Figure 1a) which is verified by the high-resolution SEM image (Figure 1b). The CS-Fe_3_O_4_ nanoparticles have an evident polymer (Figure 1c) coating with an approximate size of 10 nm (Figure 1d).

Figure 1 shows the morphology of the Fe_3_O_4_ and CS-Fe_3_O_4_ nanoparticles. The SEM image in Figure 1a depicts the morphology of the synthesized Fe_3_O_4_ nanoparticles which shows an apparent resemblance to small balls aggregation. This is further confirmed by the high-resolution SEM imaging shown in Figure 1b, which also shows the state of aggregation by small balls. In Figure 1c, the SEM image of the CS-Fe_3_O_4_ nanoparticles can be seen with evident polymer covering. In Figure 1d, the CS-Fe_3_O_4_ nanoparticles exhibit evidence the covering of CS and the nanoparticles are ∼10 nm, and therefore, it can be inferred that the CS can effectively isolate the Fe_3_O_4_ nanoparticles.

### Fourier transform infrared spectroscopy, X-ray photoelectron spectroscopy, thermogravimetric analysis, and differential scanning calorimetry analyses

**FIGURE 2:**
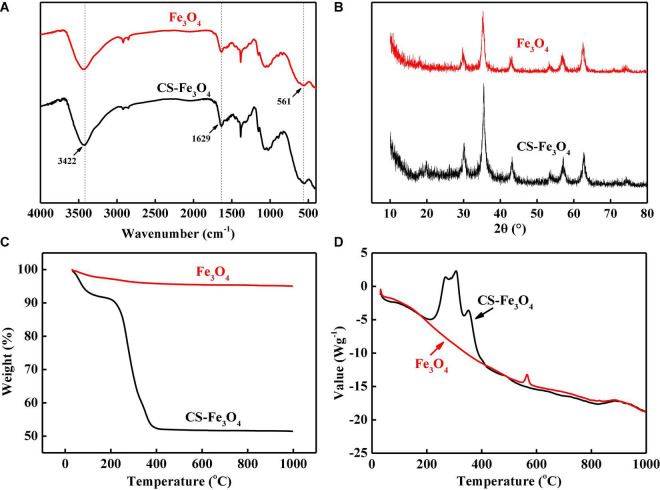
**(A)** Fourier transform infrared (FT-IR) spectra. **(B)** High-resolution X-ray diffraction (XRD) spectra of C 1s signals. **(C)** Thermogravimetric analysis (TG) curves. **(D)** Differential scanning calorimetry (DSC) curves of CS-Fe_3_O_4_ nanoparticles.

The infrared spectrum peaks at 3,422 and 1,629 cm^–1^ in Fe_3_O_4_ and CS-Fe_3_O_4_ nanoparticles are attributed to the O-H stretching vibration of water and the H-O-H bending vibration of water, respectively (Figure 2A) (41). The peak at 561 cm^–1^ is attributed to Fe_3_O_4_ (41). The diffraction peaks observed in the CS-Fe_3_O_4_ composite in Figure 2B (2θ = 30.1°, 35.5°, 42.9°, 53.3°, 57.1°, and 62.7°) were assigned to the following diffraction planes of the cubic spinel crystal structure of Fe_3_O_4_, respectively: (220), (311), (400), (422), (511), and (440) (JCPDS file no. 19-0629). The above data demonstrate that Fe_3_O_4_ in the CS-Fe_3_O_4_ composite had the same physical and chemical properties as standard Fe_3_O_4_. CS-Fe_3_O_4_ nanoparticles contained approximately 43.5% of CS and had a sharp weight loss between 215 and 397°C by TGA (Figure 2C), which is attributed to CS decomposition. The TG curve became stable at 420°C with 51.5% solid residue remaining at 1,000°C. Meanwhile, Fe_3_O_4_ nanoparticles showed a slight weight decrease between 30 and 1,000°C with 95% solid residue remaining at 1,000°C. The exothermic range of CS-Fe_3_O_4_ nanoparticles observed at 215–411°C by DSC (Figure 2D) confirmed that they started to react with the air and decomposed from approximately 215°C, followed by a sharper and stronger decomposition at ∼308°C. Meanwhile, Fe_3_O_4_ nanoparticles had a stable endothermic DSC curve except for a small exothermic peak at 564°C. In conclusion, the TG and DSC results proved that the CS-Fe_3_O_4_ nanoparticles contained abundant CS.

### X-ray photoelectron spectroscopy analysis

**FIGURE 3:**
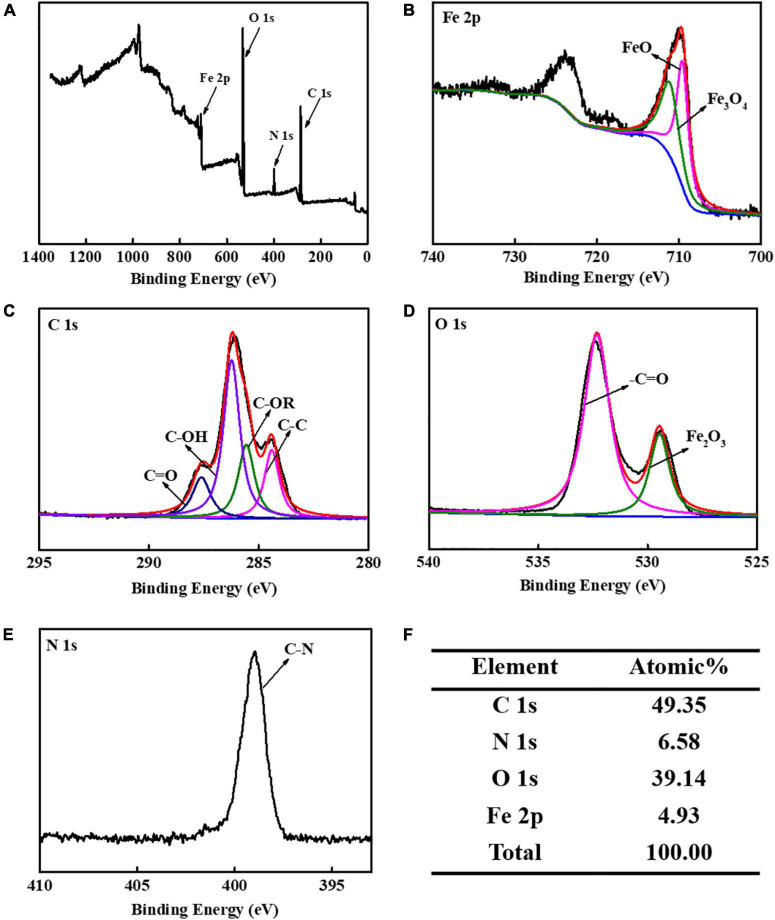
X-ray photoelectron spectroscopy (XPS) analysis of CS-Fe_3_O_4_ nanoparticles. **(A)** XPS profile. **(B)** High-resolution XPS of Fe 2p. **(C)** High-resolution XPS of C 1s signal. **(D)** High-resolution XPS of O 1s signal. **(E)** High-resolution XPS of N 1s signal. **(F)** Elemental atomic percentages of CS-Fe_3_O_4_ nanoparticles.

The CS-Fe_3_O_4_ nanoparticles have evident C 1s, N 1s, O 1s, and Fe 2p signals (Figure 3A). The high-resolution XPS of Fe 2p was found in two groups: Fe_3_O_4_ 711.3 eV and FeO 709.6 eV (Figure 3B). The fitted C 1s peaks of C=O, C–OH, C-OR, and C-C are observed at 287.6, 286.2, 285.6, and 284.4 eV, respectively, which is within 0.2 of the standard peaks: 287.8, 286.4, 285.4, and 284.6 eV, respectively (Figure 3C). The O 1s fitted peaks at 532.3 and 529.5 eV are attributed to –C=O and Fe_2_O_3_, respectively (Figure 3D), confirming that synthesized CS-Fe_3_O_4_ nanoparticles contain CS and Fe_2_O_3_. The peak at 398.9 eV attributed to C-N (Figure 3E) supported the presence of CS in CS-Fe_3_O_4_ nanoparticles. The C 1s signal occupies 49.35% of the atomic composition showing that CS efficiently covers the Fe_3_O_4_ nanoparticles (Figure 3F).

### Cell viability

**FIGURE 4:**
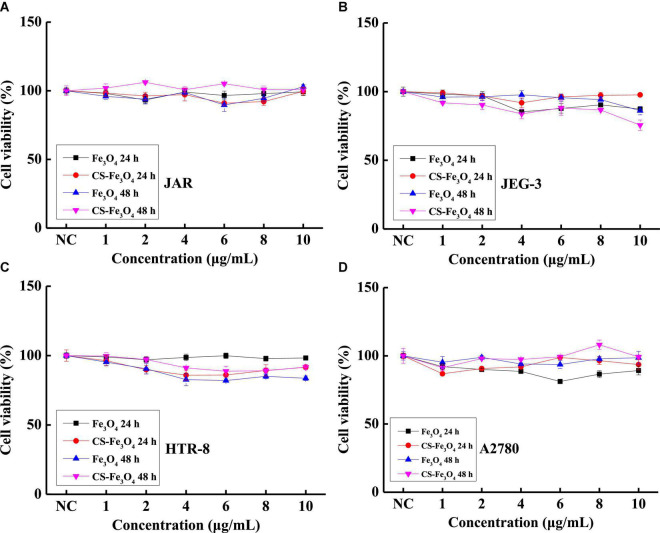
Cell viability of **(A)** JAR cells, **(B)** JEG-3 cells, **(C)** HTR-8 cells, and **(D)** A2780 cells with Fe_3_O_4_ and CS-Fe_3_O_4_ nanoparticles under different concentrations and treatment periods.

**FIGURE 5:**
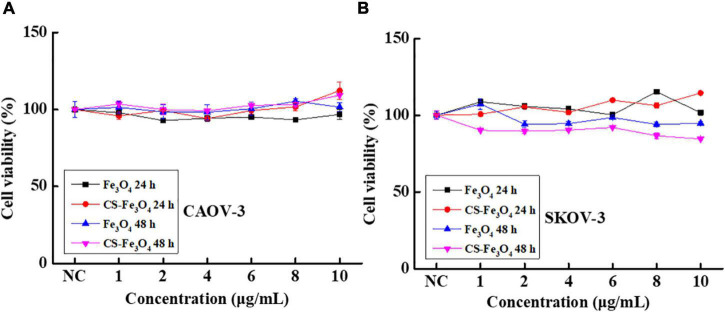
Cell viability of **(A)** CAOV-3 cells and **(B)** SKOV-3 cells with Fe_3_O_4_ and CS-Fe_3_O_4_ nanoparticles under different concentrations and treatment periods.

The cell viabilities of JAR (Figure 4A), JEG-3 (Figure 4B), HTR-8 (Figure 4C), and A2780 (Figure 4D) cells were measured after being incubated with various concentrations of the CS-Fe_3_O_4_ nanoparticles for 24 and 48 h. Figure 4 shows that the CS-Fe_3_O_4_ nanoparticles have good biocompatibility for gynecological cells. The cell viabilities of cancer cells (JAR, JEG-3, HTR-8, and A2780 cells) were estimated to be more than 80% when the concentrations were increased to 10 μg/ml, and the normal cell viabilities of HTR-8 (Figure 4C) were estimated to be more than 83% when the concentrations were increased to 10 μg/ml. For the same condition, no obvious cell viability change was observed in CAOV-3 cells (Figure 5A). There was a slight decrease in the viability of SKOV-3 cells at 48 h (Figure 5B). These results indicate distinctive cell growth, and a minimal toxicity effect can be achieved with the nanoparticles. The above data revealed that the Fe_3_O_4_ and CS-Fe_3_O_4_ nanoparticles all have good cytocompatibility.

**FIGURE 6:**
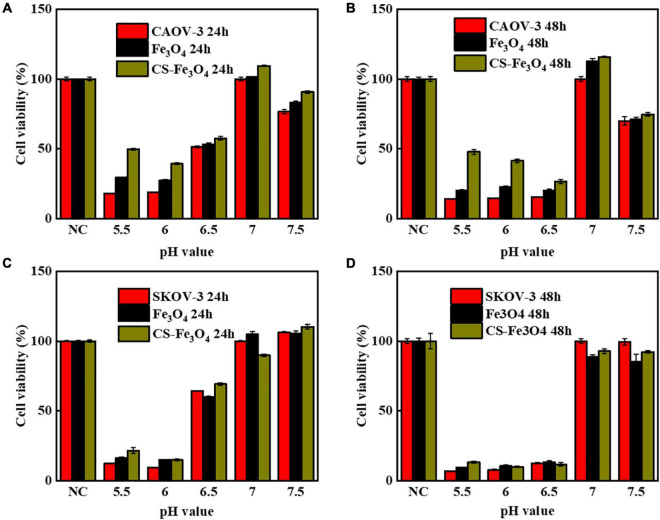
Cell viability of **(A)** CAOV-3 cells treated for 24 h, Fe3O4 treated for 24 h, and CS-Fe3O4 treated for 24 h; **(B)** CAOV-3 cells treated for 48 h, Fe3O4 treated for 48 h, and CS-Fe3O4 treated for 48 h; **(C)** SKOV-3 cells treated for 24 h, Fe3O4 treated for 24 h, and CS-Fe3O4 treated for 24 h; **(D)** SKOV-3 cells treated for 48 h, Fe3O4 treated for 48 h, and CS-Fe3O4 treated for 48 h at different pH values.

All the above-mentioned results indicate that, in normal conditions, Fe_3_O_4_ and CS-Fe_3_O_4_ nanoparticles have good cytocompatibility. The cytotoxicity test results showed that the Fe_3_O_4_ and CS-Fe_3_O_4_ nanoparticles had a strong increasing effect on cell viability under different pH value conditions, and that the cell survival rate decreased significantly as the pH value decreased (Figure 6). The CAOV-3 and SKOV-3 cells had high cell viability at pH values 6.5, 7, and 7.5 after 24 h, but the cell viability at pH value 6.5 was obviously decreased at 48 h. The survival rate of the CAOV-3 (Figure 6A) and SKOV-3 (Figure 6C) cells were below 20% at pH 5.5 and pH 6 after 24 h, and the cell viability was further decreased at 48 h (Figures 6B,D). The above results revealed that an acid environment considerably impacts cell viability, and that CS-Fe_3_O_4_ nanoparticles can efficiently improve cell viability in an acidic environment.

**FIGURE 7:**
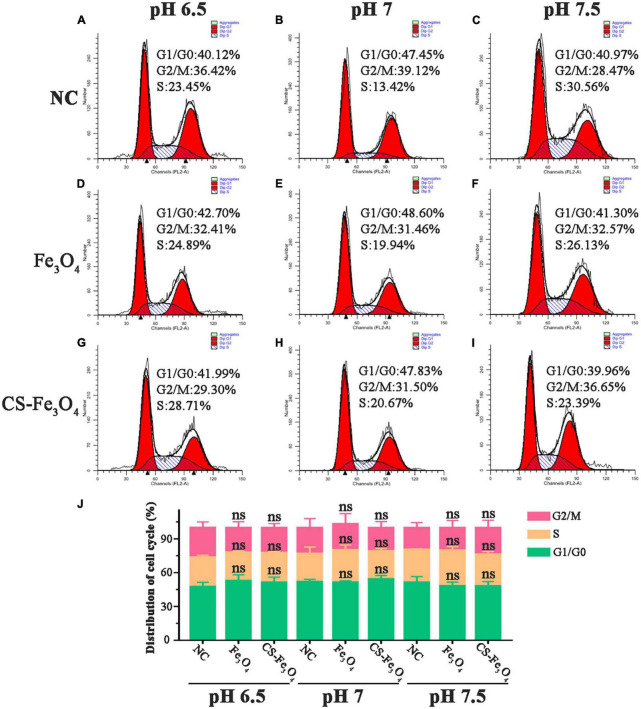
Cell flow cytometry analysis of SKOV-3 cells **(A–C)** NC, **(D–F)** Fe_3_O_4_ nanoparticles, and **(G–I)** CS-Fe_3_O_4_ nanoparticles at different pH values 24 h after treatment. **(J)** The cell cycle distributions were analyzed in the histogram. ns: no statistical difference.

The results of the MTT test revealed that the cell viability of SKOV-3 cells varied in Fe_3_O_4_ and CS-Fe_3_O_4_ treatments under acidic environments. Therefore, we hypothesized that Fe_3_O_4_ and Cs-Fe_3_O_4_ may influence gynecology cell proliferation under a lower pH level. Therefore, the cell cycle was detected using flow cytometry to observe the cell viability of SKOV-3 cells at different pH values after 24 h. As shown in Figures 7A–C, the cell cycle of SKOV-3 cells was identified in the NC group. The percentage of cell cycle SKOV-3 cells following the addition of Fe3O4 (Figures 7D–F) and CS-Fe3O4 (Figures 7G–I) nanoparticles was stable (10 μg/ml) at different pH values. Compared to the NC group under pH 6.5 and pH 7, neither Fe_3_O_4_ nor CS-Fe_3_O_4_ caused phase arrest in SKOV-3 cells after 24 h of exposure (Figure 7J). In addition, there were no apoptotic changes, such as cell shrinkage, in SKOV-3 cells treated for 24 h with Fe_3_O_4_ or CS-Fe_3_O_4_ (10 μg/ml), as identified using EDU staining (Supplementary Figure 4). Of course, there were no statistical differences in the cell cycle at different pH values. This indicates that Fe_3_O_4_ and CS-Fe_3_O_4_ do not play a role in cell cycles at low pH levels.

**FIGURE 8:**
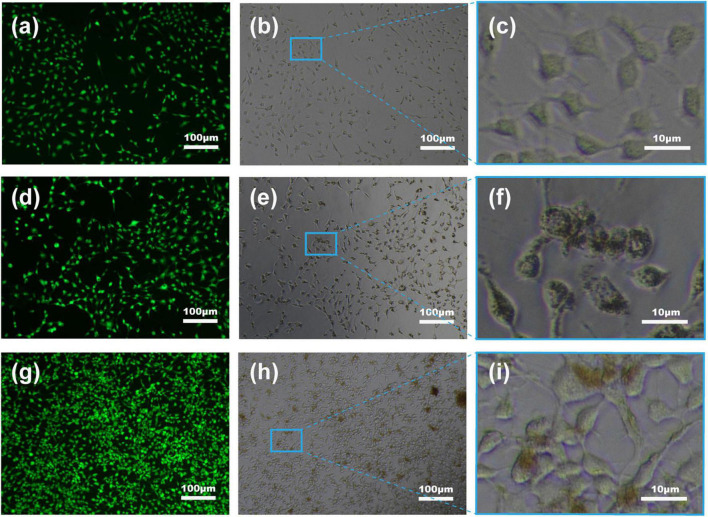
Fluorescent images of SKOV-3 cells following staining with calcein AM. **(a–c)** NC, **(d–f)** Fe_3_O_4_ nanoparticles, and **(g–i)** CS-Fe_3_O_4_ nanoparticles at pH 7 after 24 h after treatment.

Untreated living cells have weak fluorescence following staining with calcein AM with only some of the non-fluorescent intracellular dye converted to a green, fluorescent substance (Ex/Em: 495/520 nm) by intracellular esterases due to partial permeability of the membrane (Figure 8a). The greater signal in Fe_3_O_4_ treatment (Figure 8d) and especially CS-Fe3O4 treatment (Figure 8g) indicates nanoparticles have facilitated a greater uptake of this dye cells compared with the NC (Figure 8c). Moreover, compared with the NC (Figure 8b), SKOV-3 cells efficiently uptake Fe_3_O_4_ and CS-Fe_3_O_4_ nanoparticles (Figures 8e,h), as shown better in the high magnification (Figures 8f,i). The addition of PI detects damaged cell membranes (or dead cells) by binding to nucleic acids and producing a bright red fluorescent signal (Ex/Em: 530/620 nm) (Supplementary Figures 1–3). The small fluorescent red dots shown in Supplementary Figures 2, 3 indicate that SKOV-3 cell death did not occur. This phagocytosis effect means that the drugs can easily enter cells when they are combined with these nanoparticles. The above data indicate that the cells had high survival rates after phagocytizing abundant quantities of Fe_3_O_4_ and CS-Fe_3_O_4_ nanoparticles.

### Relevant mechanisms

**FIGURE 9:**
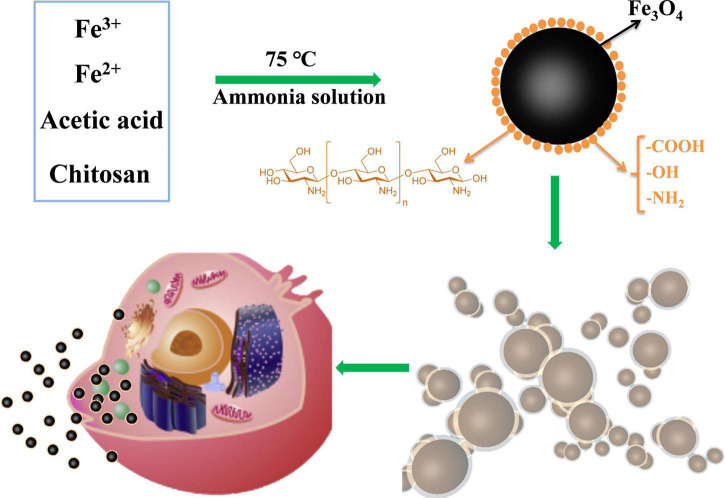
Schematic diagram showing the preparation of CS-Fe_3_O_4_. The surface functional groups of chitosan-wrapped Fe_3_O_4_ nanoparticles improve their cytocompatibility.

Chitosan (CS) has strong intramolecular and intermolecular hydrogen bonding interactions in its structure; therefore, it cannot be dissolved in water (35, 38). In this work, one % w/v acetic acid was used to dissolve CS, followed by the addition of Fe^3+^ and Fe^2+^ (Figure 9). Fe_3_O_4_ was formed when the ammonia solution was added at 75°C, and CS was simultaneously recrystallized because of the acid loss. The CS-Fe_3_O_4_ nanoparticles prepared using this method are smaller than those produced *via* the conventional co-precipitation method and are expected to improve the deposition of medicinal drugs. Engulfment of Fe_3_O_4_ molecules by CS prevents agglomeration between nanoparticles, and the biocompatible CS surface is beneficial to cytophagocytosis (Figure 9). The CS-Fe_3_O_4_ nanoparticles have better biodegradability and biocompatibility than Fe_3_O_4_ because the organic surface has abundant functional groups that can deposit various materials.

## Conclusion

In this experiment, the sol-gel method was successfully used to prepare Fe_3_O_4_ nanoparticles, and CS was used to engulf Fe_3_O_4_ nanoparticles. The CS-Fe_3_O_4_ nanoparticles were characterized by SEM, TEM, FT-IR, XPS, TG, and DSC and compared with Fe_3_O_4_. The CS-Fe_3_O_4_ nanoparticles are biocompatible with gynecological cells *in vitro* and exhibit higher viability than Fe_3_O_4_ nanoparticles at different pH values. CS-Fe_3_O_4_ nanoparticles phagocytized by gynecological cells do not affect cell viability. These properties favor CS-Fe_3_O_4_ nanoparticles for the deposition of various materials such as drugs in cellular compartments. Further *in vivo* studies are required to determine the applicability of this nanoparticle for cell targeting, hyperthermia therapy, and medical imaging.

## Data availability statement

The original contributions presented in the study are included in the article/Supplementary Material, further inquiries can be directed to the corresponding authors.

## Author contributions

All authors discussed the results and commented on the manuscript, contributed to the article, and approved the submitted version.

## Conflict of interest

The authors declare that the research was conducted in the absence of any commercial or financial relationships that could be construed as a potential conflict of interest.

## Publisher’s note

All claims expressed in this article are solely those of the authors and do not necessarily represent those of their affiliated organizations, or those of the publisher, the editors and the reviewers. Any product that may be evaluated in this article, or claim that may be made by its manufacturer, is not guaranteed or endorsed by the publisher.

## Supplementary material

Supplementary Figure 1 Red fluorescent signal of untreated SKOV-3 cells.

Supplementary Figure 2 Red fluorescent signal of SKOV-3 cells with 10 μg/ml Fe_3_O_4_ nanoparticles.

Supplementary Figure 3 Red fluorescent signal of SKOV-3 cells with 10 μg/ml CS-Fe_3_O_4_ nanoparticles.

Supplementary method: 2.5.4 EDU cell proliferation assay: The effect of Fe_3_O_4_ and CS-Fe_3_O_4_ on SKOV-3 cell proliferation was also measured by EDU staining (BeyoClick EdU Cell Proliferation Kit with Alexa Fluor 488, Beyotime, China) incorporation assay as recommended by the manufacturer.

Supplementary Figure 4 The cell viability effects of Fe_3_O_4_ and CS-Fe_3_O_4_ nanoparticles with different pH value on SKOV-3 cells for 24 h through EDU staining.
