# Supplementary figures and images for: Cytocompatibility of pH-sensitive, chitosan-coated Fe3O4 nanoparticles in gynecological cells
Source: Front Med (Lausanne). 2022 Jul 22;9:799145. doi: 10.3389/fmed.2022.799145 (PMC9355084; doi:10.3389/fmed.2022.799145)

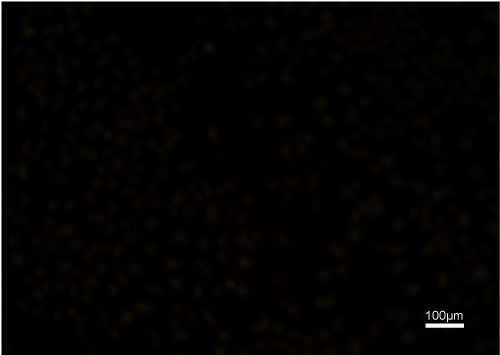

Supplement: Supplementary Figure 1 — Red fluorescent signal of untreated SKOV-3 cells. [file Image_1.TIF]

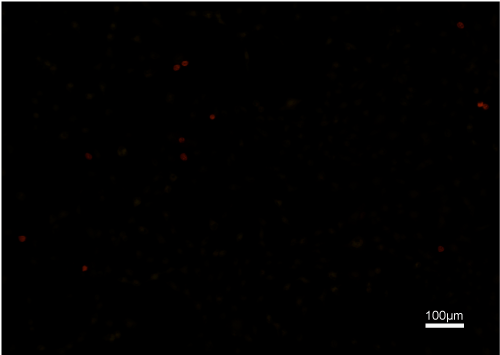

Supplement: Supplementary Figure 2 — Red fluorescent signal of SKOV-3 cells with 10 μg/ml Fe3O4 nanoparticles. [file Image_2.TIF]

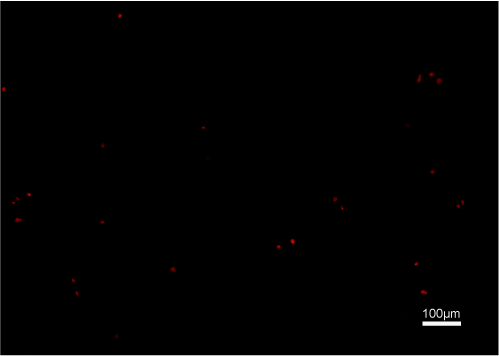

Supplement: Supplementary Figure 3 — Red fluorescent signal of SKOV-3 cells with 10 μg/ml CS-Fe3O4 nanoparticles. [file Image_3.TIF]

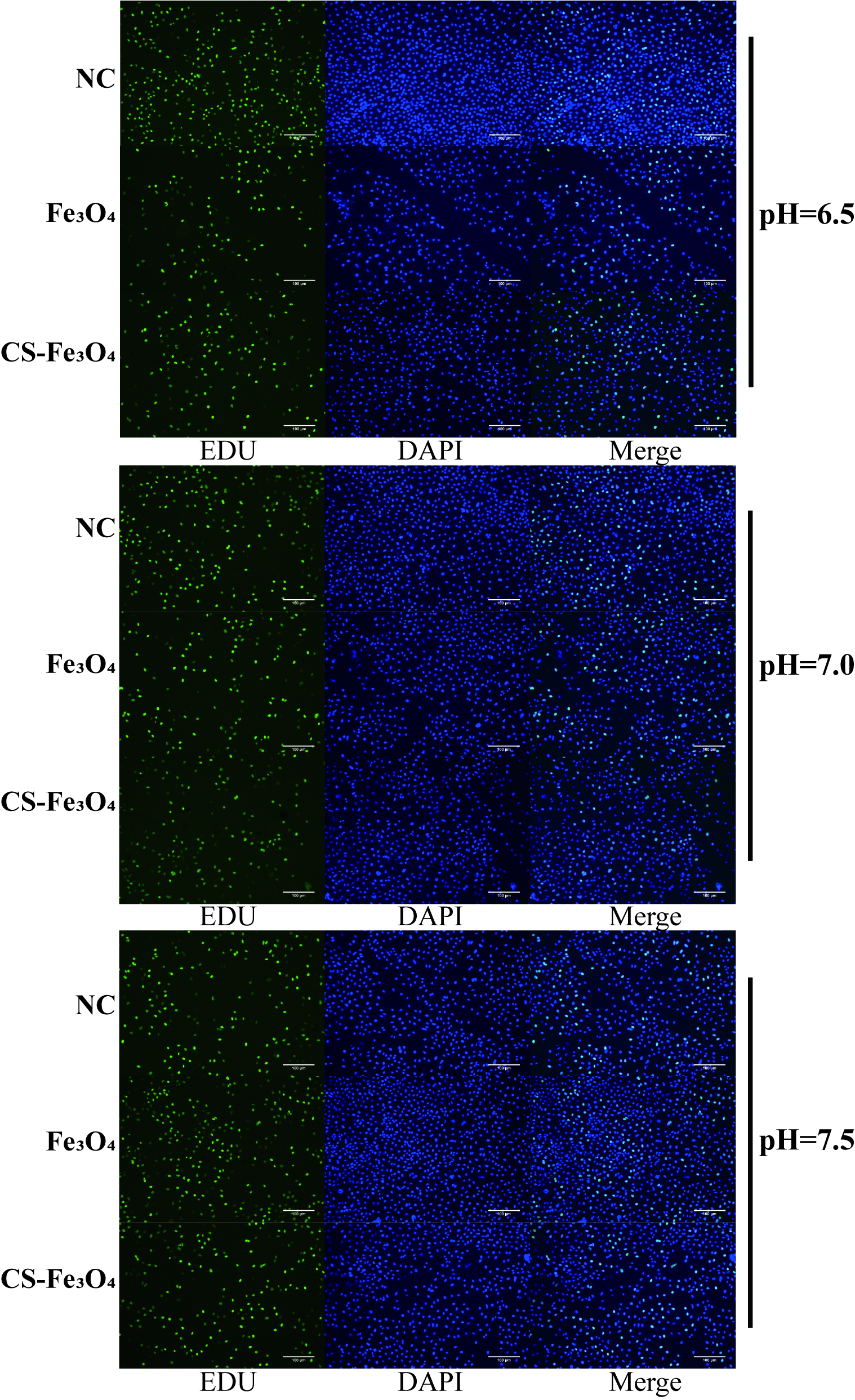

Supplement: Supplementary method: 2.5.4 — EDU cell proliferation assay: The effect of Fe3O4 and CS-Fe3O4 on SKOV-3 cell proliferation was also measured by EDU staining (BeyoClick EdU Cell Proliferation Kit with Alexa Fluor 488, Beyotime, China) incorporation assay as recommended by the manufacturer. [file Image_4.TIF]
